# Causal effects of gut microbiota on multiple sclerosis: A two‐sample Mendelian randomization study

**DOI:** 10.1002/brb3.3593

**Published:** 2024-06-19

**Authors:** Dongren Sun, Yangyang Zhang, Rui Wang, Qin Du, Ziyan Shi, Hongxi Chen, Xiaofei Wang, Hongyu Zhou

**Affiliations:** ^1^ Department of Neurology West China Hospital Sichuan University Chengdu China

**Keywords:** causal effect, gut microbiota, Mendelian randomization, multiple sclerosis

## Abstract

**Background:**

Gut microbiota alterations in multiple sclerosis (MS) patients have been reported in observational studies, but whether these associations are causal is unclear.

**Objective:**

We performed a Mendelian randomization study (MR) to assess the causal effects of gut microbiota on MS.

**Methods:**

Independent genetic variants associated with 211 gut microbiota phenotypes were selected as instrumental variables from the largest genome‐wide association studies (GWAS) previously published by the MiBioGen study. GWAS data for MS were obtained from the International Multiple Sclerosis Genetics Consortium (IMSGC) for primary analysis and the FinnGen consortium for replication and collaborative analysis. Sensitivity analyses were conducted to evaluate heterogeneity and pleiotropy.

**Results:**

After inverse‐variance‐weighted and sensitivity analysis filtering, seven gut microbiota with potential causal effects on MS were identified from the IMSGC. Only five metabolites remained significant associations with MS when combined with the FinnGen consortium, including genus *Anaerofilum id.2053* (odds ratio [OR] = 1.141, 95% confidence interval [CI]: 1.021–1.276, *p* = .021), *Ruminococcus2 id.11374* (OR = 1.190, 95% CI: 1.007–1.406, *p* = .042), *Ruminococcaceae UCG003 id.11361* (OR = 0.822, 95% CI: 0.688–0.982, *p* = .031), *Ruminiclostridium5 id.11355* (OR = 0.724, 95% CI: 0.585–0.895, *p* = .003), *Anaerotruncus id.2054* (OR = 0.772, 95% CI: 0.634–0.940, *p* = .010).

**Conclusion:**

Our MR analysis reveals a potential causal relationship between gut microbiota and MS, offering promising avenues for advancing mechanistic understanding and clinical investigation of microbiota‐mediated MS.

## Graphical abstract

IVs: instrumental variables; SNP: single nucleotide polymorphism; MR: Mendelian randomization; ↑: adverse effect; ↓: protective effect.

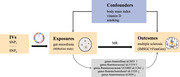

## 1 INTRODUCTION

Multiple sclerosis (MS) is a chronic inflammatory neurological disease of the brain and spinal cord characterized by inflammation, demyelination, and subsequent neuronal loss, affecting an estimated 2.5 million people worldwide (Compston & Coles, 2008; Dendrou et al., 2015). Over the past 20 years, immunomodulatory treatments for MS patients have received considerable attention, with several encouraging results in clinical practice. These findings highlight the link between relapses and progression of MS and various immune pathways (Dendrou et al., 2015). Recent evidence suggests a functional alteration of regulatory T cells (Tregs) and/or effector B and T cells. Such perturbations compromise peripheral tolerance mechanisms, leading to the activation of peripheral autoreactive B and T cells. As a result, cellular entities, including CD8^+^ T cells, CD4^+^ T helper (TH) 1 cells, TH17 cells, and B cells, breach the blood‐brain barrier and infiltrate the central nervous system (CNS). This infiltration promotes the activation of both microglia and astrocytes, culminating in extensive demyelination and axonal injury (Bielekova et al., 2004; Cosorich et al., 2017; Dendrou et al., 2015; Patrick et al., 2023).

The complex relationship between the gut microbiome and human immune responses is becoming increasingly clear, particularly the role of the gut‐brain axis in the development of MS (Correale et al., 2022). Both the composition of the gut microbiota and the integrity of the intestinal barrier are central players in this axis (Ghezzi et al., 2021). Under typical conditions, signaling from the gut microbiota involves intricate communication among various cells, including dendritic cells, macrophages, and lymphocytes, orchestrating a myriad of physiological processes. Both the innate and adaptive immune systems intricately modulate these processes (Correale et al., 2022; Parodi & Kerlero de Rosbo, 2021). Dysbiosis, or an imbalance in the gut microbiota, can suppress Tregs and promote the proliferation of inflammatory T cells, such as TH1 or TH17 cells. This leads to an immune imbalance and a systemic inflammatory response that further accelerates the progression of MS. Conversely, a balanced or specific microbial composition may slow the development of MS (Correale et al., 2022; Ghezzi et al., 2021). Several studies have supported this view. For example, certain commensal bacterial species, including *Escherichia coli*, *Bifidobacterium adolescentis*, *Candida albicans*, and *Lactobacillus*, have been identified as modulators in promoting or suppressing the production of Tregs and inflammatory T cells (TH1 or TH17 cells) (Cosorich et al., 2017; Geuking et al., 2011; Honda & Littman, 2016). In addition, an excess of TH17 cells in the gut has been associated with increased disease activity in MS patients (Cosorich et al., 2017).

Alterations in the composition of the gut microbiome in MS patients have been widely reported (Cantarel et al., 2015; Johanson et al., 2020). However, there are notable inconsistencies in these findings. While the majority of studies suggest a decrease in *Ruminococcaceae* in MS patients (Tremlett et al., 2016, 2021), Galluzzo et al. (2021) documented an increased abundance of *Ruminococcaceae* in MS patients compared to healthy controls. In addition, studies in experimental autoimmune encephalomyelitis (EAE) mice revealed an increased abundance of *Ruminococcaceae* during critical stages of EAE progression (Johanson et al., 2020). Accumulating evidence points to butyrates as potential suppressors of MS onset and progression (Chen et al., 2019; Horton et al., 2021). Since *Anaerotruncus* is a known butyrate producer, it should theoretically reduce the risk of MS when present in the gut microbiome. Surprisingly, a rodent study showed that *Anaerotruncus* exacerbated MS progression (Bianchimano et al., 2022). These inconsistencies are largely because most studies are observational and focus on the gut microbiome of already diagnosed MS patients, making it difficult to discern true exposures and outcomes. Another important consideration is that the association between the gut microbiome and MS could be confounded by various factors such as diet, medication use, and environmental exposures (Rinninella et al., 2019).

Establishing an economically efficient method to unravel the causal relationship between the gut microbiome and MS could pave the way to discovering potential microbiome‐based therapeutic avenues for MS. Theoretically, conducting randomized controlled trials of the gut microbiome or conducting large‐scale prospective cohort studies seems plausible. However, challenges related to cost, design, and other factors make such approaches difficult to execute. Coincidentally, genome‐wide association studies (GWAS) have made significant advances in the last decade as a tool for delineating associations between genotypes and phenotypes (Ishigaki, 2022). Mendelian randomization (MR) makes powerful use of GWAS data to establish causal relationships between exposures and outcomes (Sun, Wang, et al., 2023; Sun, Du, et al., 2023; Sun, Tu, et al., 2023; Wang et al., 2022). The accumulating body of MR studies has been instrumental in resolving ongoing epidemiologic debates, particularly in MS, and has served as a major catalyst for progress in neurology (Adams et al., 2024; Gagnon et al., 2024; Vandebergh et al., 2022). Utilizing a two‐sample MR framework, these studies use different exposure and outcome samples for causal inference, eliminating the need to monitor disease dynamics within the same cohort as required in observational studies to delineate risk factors (Burgess et al., 2019). Because genetic variants are randomly assigned at birth and remain unchanged with disease onset or progression, MR effectively circumvents the inherent shortcomings of observational studies, such as reverse causation and confounding (Yuan & Larsson, 2022).

Here, we aim to use the largest available GWAS dataset to elucidate the causal role of the gut microbiome in MS, providing new insights into its pathogenesis and potential treatments.

## 2 METHODS

### 2.1 Study design

**FIGURE 1:**
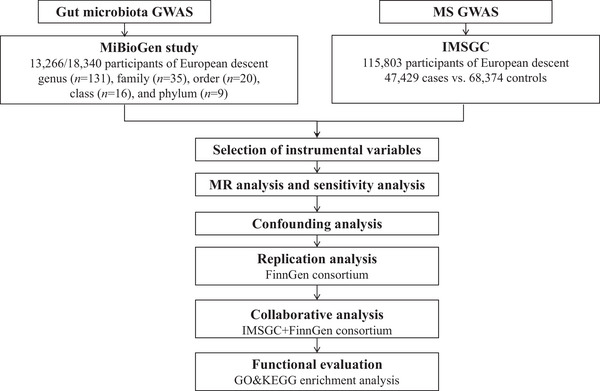
General flowchart of the study. Here, a two‐sample mendelian randomization (MR) framework comprehensively assessed the causal effects of exposure (gut microbiota) on the outcome multiple sclerosis (MS). Filtered MR results were replicated and subjected to collaborative analysis by the FinnGen consortium. Finally, integrated causal gene gene ontology (GO) and Kyoto Encyclopedia of Genes and Genomes (KEGG) enrichment analyses revealed biological pathways through which gut microbiota may act on MS. GWAS, genome‐wide association studies; IMSGC, International Multiple Sclerosis Genetics Consortium.

The methodology of our study is outlined in Figure 1. We employed the two‐sample MR method to systematically evaluate the causal associations of 211 gut microbiota taxa with MS. Replication analysis was subsequently undertaken within the FinnGen consortium, followed by a collaborative analysis that integrated MR estimates from both datasets. To investigate potential pathways through which the gut microbiota might modulate MS, we conducted an enrichment analysis based on causal genes. Specifically, we implemented Bonferroni corrections based on the counts of various gut microbiota taxa categories, leading to computed significance thresholds as: genus: 3.81 × 10^−4^ (0.05/131), family: 1.4 × 10^−3^ (0.05/35), order: 2.5 × 10^−3^ (0.05/20), class: 3.1 × 10^−3^ (0.05/16), and phylum: 5.5 × 10^−3^ (0.05/9). A threshold of *p* < .05 was set for nominal significance. All analyses were executed using R software (version 4.2.1), and its respective packages: TwoSampleMR (version 0.5.6), meta (version 6.2‐1), and clusterProfiler (version 4.4.4) complemented by the online platform at https://www.bioinformatics.com.cn. Our statistical framework strictly adhered to the STROBE‐MR guidelines (Skrivankova et al., 2021), with the analysis being conducted from October 2022 to March 2023.

### 2.2 Data sources

Building on previous MR studies (Li et al., 2022; Li et al., 2023), we have accessed data from the MiBioGen study, which includes 211 species of gut flora GWAS data (Kurilshikov et al., 2021). This extensive database, which represents the most comprehensive collection to date, not only enhances the robustness of MR analysis, but also provides a diverse array of microbial species for comprehensive evaluation of their influence on MS (Burgess et al., 2019). Specifically, these microbiota taxa are categorized into five groups: genus (*n* = 131), family (*n* = 35), order (*n* = 20), class (*n* = 16), and phylum (*n* = 9). The MiBioGen study integrates 24 cohorts comprising 18,340 participants, of which 13,266 individuals (from 16 cohorts) are of European ancestry. Utilizing participants' 16S rRNA gene sequencing profiles and genotypic data, the study identified 31 genetic loci influencing the microbiome composition, reaching genome‐wide significance thresholds (*p* < 5×10^−8^) (Kurilshikov et al., 2021). Our outcome GWAS summary statistics for the MS phenotype were derived from the recently published GWAS meta‐analysis by the International Multiple Sclerosis Genetics Consortium (IMSGC), encompassing 47,429 cases versus 68,374 controls. This extensive GWAS meta‐analysis accounts for up to 48% of the heritability estimate for MS and pinpoints potential functional outcomes of specific MS loci (International Multiple Sclerosis Genetics Consortium, 2019). Researchers have identified 551 prioritized susceptibility genes for MS that are particularly associated with several brain‐resident immune cells, particularly natural killer cells and dendritic cells. In addition, they found enrichment of these gene sets in microglia rather than astrocytes, highlighting the potential involvement of brain‐resident immune cells in the pathogenesis of MS (International Multiple Sclerosis Genetics Consortium, 2019). For validation, we leveraged MS data from the FinnGen consortium (GWASID: finn‐b‐G6_MS), which comprises 1048 patients and 217,141 controls of European descent across 16,380,460 single nucleotide polymorphisms (SNPs) (Kurki et al., 2023). Specifically, MS diagnoses in Finland are based on nationwide health registers using ICD‐10 code G35, ICD‐9 code 340, and ICD‐8 code 34099. The mean age at the first MS event is 39.78 years, with a 5‐year case fatality rate of 1.05% (Kurki et al., 2023).

### 2.3 Selection of instrumental variables

First, to ensure sufficient instrumental variables (IVs), we relaxed the significance threshold for IVs to *p* < 1E‐5. Second, using the European 1000 Genomes Project Phase 3 as a reference panel, we adjusted the clump settings to *R* ^2^ < 0.01 and a distance of 1000 kb to assess the effect of linkage disequilibrium (LD) among the included SNPs. Third, we extracted information corresponding to the IVs of the outcome trait. In the absence of consistent SNPs, proxy SNPs were used with an LD threshold set between 0.8 and 1. Fourth, palindromic SNPs were excluded to ensure consistency in allele coding or strand orientation between exposure and outcome IVs. Fifth, we calculated the *F*‐statistic for each IV using the formula *F* = beta^2^/se^2^ to reduce bias from weak instruments (Burgess & Thompson, 2011; Harroud et al., 2021). Only SNPs with an *F*‐statistic >10 were retained for subsequent MR analysis.

### 2.4 MR analysis and sensitivity analysis

The multiplicative random‐effects inverse‐variance‐weighted (IVW) approach was used as the primary analysis to determine the causal relationship between gut microbiota and MS. The IVW method assumes that all included genetic variations are valid instruments and synthesizes the Wald ratios of all IVs by meta‐analysis to estimate the effect of gut microbiota on MS. Assuming no horizontal pleiotropy, IVW estimates would be unbiased. In addition, the random effects IVW approach account for the presence of heterogeneity (Burgess et al., 2017). The MR‐Egger, weighted median, simple mode, and weighted mode methods serve as supplements to the IVW method (Bowden et al., 2016) and further assess the robustness of MR estimates. The Cochran *Q* test was used to measure the degree of heterogeneity among the individual effect estimates derived from each IV (Haycock et al., 2016). Significant non‐zero intercepts in the Egger intercept test (Bowden et al., 2015) and a *p*‐value threshold of <.05 in the MR‐PRESSO global test (Verbanck et al., 2018) suggest the presence of horizontal pleiotropy. In addition, the MR‐PRESSO method can detect potential outliers by providing corrections for horizontal pleiotropy in MR estimates (Sun, Du, et al., 2023; Verbanck et al., 2018).

### 2.5 Confounding analysis

Although several sensitivity analyses were employed to gauge the robustness of our MR findings, potential confounding could undermine MR estimates. Research indicates that body mass index (BMI), vitamin D, and smoking may act as risk factors for MS (Olsson et al., 2017; Wesnes et al., 2021). Consequently, we used the PhenoScanner tool to filter out SNPs reaching significance thresholds associated with these confounders and the outcome (*p* < 1E‐5) and subsequently recalibrated our MR estimates (Kamat et al., 2019).

### 2.6 Replication and collaborative analysis

To further substantiate our findings, we used the MS dataset from the FinnGen consortium (Kurki et al., 2023) to replicate our analyses using the IVW method. Subsequently, a collaborative analysis of the two MR estimates for the same gut microbiota was performed to identify the final candidates.

### 2.7 Functional evaluation of causal gene panel

To delineate the potential mechanisms by which genetic proxies of the gut microbiota mediate the onset of MS, causal gene sets were derived based on collaborative analysis selections from five MR estimates. Both gene ontology and Kyoto Encyclopedia of Genes and Genomes enrichment analyses were used to identify candidate pathways involved in gut microbiota involvement in MS.

## 3 RESULTS

### 3.1 MR analysis

**FIGURE 2:**
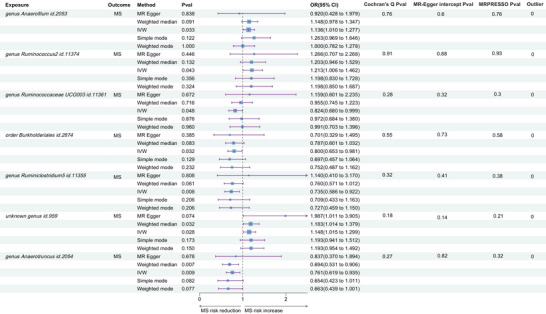
Causal estimation and sensitivity analysis of gut microbiota in multiple sclerosis. MRPRESSO Pval: *p*‐value for the mendelian randomization (MR) pleiotropy residual sum and outlier global test. Inverse‐variance‐weighted (IVW): the multiplicative random‐effects IVW approach. Among the 211 gut microbiota species, the IVW method identified seven gut microbiota species with nominally significant MR estimates for multiple sclerosis (obtained from the International Multiple Sclerosis Genetics Consortium). *p*‐values from Cochran's *Q*, MR‐Egger intercept, and MR‐PRESSO tests were all >.05, indicating that these MR estimates are robust. CI, confidence interval; IMSGC: International Multiple Sclerosis Genetics Consortium; MS, multiple sclerosis; OR, odds ratio.

Of the 211 MR analyses performed, all SNPs had an *F*‐statistic >10, indicating the absence of weak instrumental bias. Using the IVW method, we identified significant causal associations between MS and seven gut microbiota taxa, as detailed in Figure 2. These gut microbiota taxa had a range of 9–12 IVs.

Indicative evidence supported the association of increased MS risk with three gut microbiota taxa: genus *Anaerofilum id.2053* (odds ratio [OR] = 1.136, 95% confidence interval [CI]: 1.010–1.277, *p* = .033), *Ruminococcus2 id.11374* (OR = 1.212, 95% CI: 1.006–1.462, *p* = .043), and unknown genus *id.959* (OR = 1.148, 95% CI: 1.015–1.299, *p* = .028). Conversely, four gut microbiota taxa showed negative associations with MS risk: *genus Ruminococcaceae UCG003 id.11361* (OR = 0.824, 95% CI: 0.681–0.999, *p* = .048), *order Burkholderiales id.2874* (OR = 0.800, 95% CI: 0.653–0.981, *p* = .032), *Ruminiclostridium5 id.11355* (OR = 0.735, 95% CI: 0.586–0.922, *p* = .008), and *Anaerotruncus id.2054* (OR = 0.761, 95% CI: 0.619–0.935, *p* = .009).

### 3.2 Sensitivity analysis and confounding analysis

As shown in Figure 2, Cochran's *Q* test did not indicate the presence of heterogeneity, with all tests yielding *p* values >.05. Although we found non‐zero intercepts in seven Egger intercept tests, none reached significance, indicating the absence of horizontal pleiotropy (all *p* > .05). Similarly, the MR‐PRESSO method did not detect potential outliers and provided no evidence for the presence of horizontal pleiotropy (all *p* > .05). In addition, we queried the PhenoScanner online platform to determine whether any IVs were associated with BMI, vitamin D, smoking, or MS. Coincidentally, no SNPs associated with these potential confounders were identified, keeping our MR estimates consistent with previous findings.

### 3.3 Replication and collaborative analysis

**FIGURE 3:**
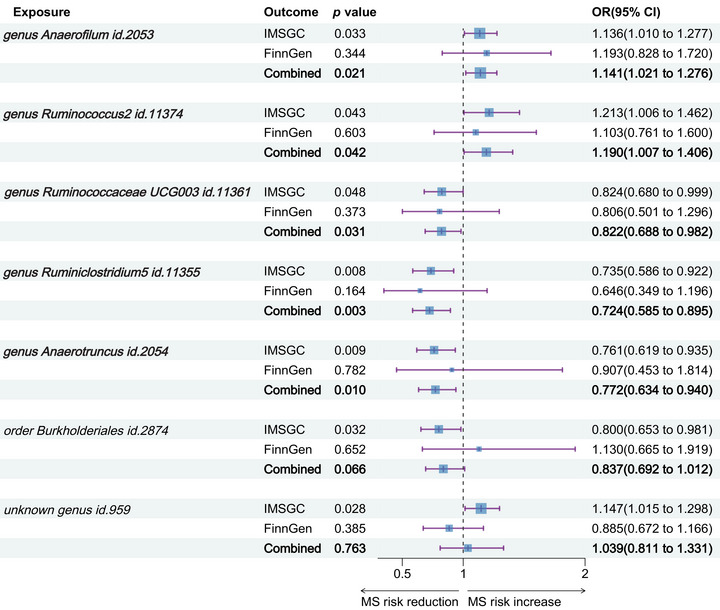
Collaborative analysis of causal relationships between gut microbiota and multiple sclerosis. We performed a joint analysis of the MR estimates obtained from the inverse‐variance‐weighted (IVW) approach derived from the International Multiple Sclerosis Genetics Consortium (IMSGC) and the FinnGen consortium, respectively. Finally, we identified five gut microbiota species that remained significant after the joint analysis (with continued significance). CI, confidence interval; OR, odds ratio.

Replication analysis using the MS data from the FinnGen consortium affirmed the directionality of IVW results for five out of the seven taxa, in line with our previous MR findings, though these did not achieve statistical significance. Merging results from the IMSGC and FinnGen consortium, we conducted a collaborative analysis. This analysis corroborated that genetically instrumented gut microbiota, specifically *genus Anaerofilum id.2053* (OR = 1.141, 95% CI: 1.021–1.276, *p* = .021), *Ruminococcus2 id.11374* (OR = 1.190, 95% CI: 1.007–1.406, *p* = .042), *Ruminococcaceae UCG003 id.11361* (OR = 0.822, 95% CI: 0.688–0.982, *p* = .031), *Ruminiclostridium5 id.11355* (OR = 0.724, 95% CI: 0.585–0.895, *p* = .003), and *Anaerotruncus id.2054* (OR = 0.772, 95% CI: 0.634–0.940, *p* = .010) have causal ties with MS. The collaborative analysis no longer substantiated a causal effect on MS for *order Burkholderiales id.2874* (OR = 0.837, 95% CI: 0.692–1.012, *p* = .066) and unknown genus *id.959* (OR = 1.039, 95% CI: 0.811–1.331, *p* = .763) (Figure 3).

### 3.4 Functional evaluation of causal genes

**FIGURE 4:**
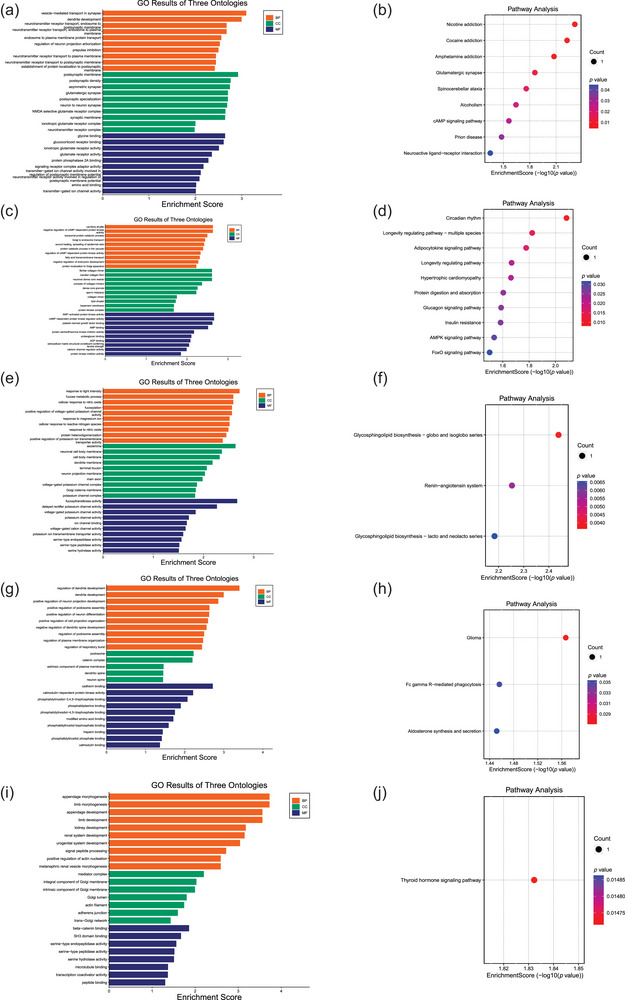
Functional evaluation of causal genes: (a, c, e, g, and i) the analysis results of gene ontology (GO); (b, d, f, h, and j) the Kyoto Encyclopedia of Genes and Genomes (KEGG) analysis results. The enrichment analysis plot of the surviving five mendelian randomization (MR) estimates revealed potential biological pathways through which gut microbiota regulate multiple sclerosis risk. BP stands for biological process, CC stands for cellular component, and MF stands for molecular function. These pathways mainly involve interactions between cell membranes, in particular synaptic and neurotransmitter transport, dendritic development, cellular oxidative stress response, and positive/negative regulation of neurons and organelles.

We ultimately identified genes that potentially mediate the causal relationship between MS and specific gut microbiota taxa: six genes for *Anaerofilum id.2053*, four for *Ruminococcus2 id.11374*, five for *Ruminococcaceae UCG003 id.11361*, seven for *Ruminiclostridium5 id.11355*, and six for *Anaerotruncus id.2054* (Table S1). Enrichment analyses suggest that the pathways involved in MS risk modulated by these gut microbiota primarily involve intercellular communication, especially synaptic transmission and neurotransmitter transport, dendritic development, cellular oxidative stress responses, and positive and negative regulation of neuronal cell organelles (Figure 4).

## 4 DISCUSSION

While previous research investigating the relationship between gut microbiota and MS has been predominantly observational (Cantarel et al., 2015; Horton et al., 2021; Tremlett et al., 2021), it remains challenging to conclusively establish the causal relationship between gut microbiota and increased or decreased risk of MS. In our current MR study, we utilized the most comprehensive GWAS datasets available and validated our findings in additional datasets. We conclusively identified genetically instrumented gut microbiota, specifically genus *Anaerofilum id.2053*, *Ruminococcus2 id.11374*, *Ruminococcaceae UCG003 id.11361*, *Ruminiclostridium5 id.11355*, and *Anaerotruncus id.2054*, as potentially causally associated with MS. These findings may provide greater objectivity and reliability compared to cross‐sectional evidence, underscoring the involvement of the gut microbiota in the pathogenesis of MS and supporting the existence of the gut‐brain axis. Characteristics of the gut microbiota serve as markers of individual immune or metabolic traits and fit well with the personalized and comprehensive treatment strategies recommended by current MS management guidelines. Targeted modulation of the gut microbiota may serve as an adjunct to disease‐modifying therapy (Kujawa et al., 2023; Olek, 2021).

Several studies have reported associations between gut microbiota and MS (Correale et al., 2022; iMSMS Consortium, 2022; Schepici et al., 2019). In our current investigation, we identified potential causal relationships between five types of gut microbes and MS, though these findings remain suggestive. Several animal models and clinical studies have suggested that *Ruminococcus* is associated with an increased risk of MS (Cantarel et al., 2015; Chen et al., 2019), which is consistent with the results of our MR analysis. Current knowledge of the role of *Ruminococcus* is limited, but it is suggested that *Ruminococcus* may be involved in oxidative stress responses, which amplify an inflammatory milieu in the gut. This leads to a shift in the T cell balance toward IL‐17 production, thereby promoting autoimmunity and inflammatory responses (Azzouz et al., 2019; Cantarel et al., 2015). Previous studies have confirmed the association of *Ruminococcus gnavus* with Crohn's disease and rheumatoid arthritis (Henke et al., 2019; Rogier et al., 2017). In addition, *Ruminococcus gnavus* levels correlate with overall disease activity in systemic lupus erythematosus and are most pronounced in patients with lupus nephritis (Azzouz et al., 2019). Notably, *Ruminococcus* has a higher number of operational taxonomic units in MS patients compared to healthy individuals. Interestingly, there's a reduction in *Ruminococcus* in MS patients treated with glatiramer acetate combined with high‐dose vitamin D supplements compared to healthy participants or untreated MS patients (Cantarel et al., 2015). It is worth noting that one particular animal study showed a reduction in *Ruminococcus* species associated with a decrease in Th17 responses and an increase in Treg responses after pharmacological intervention in a mouse model of EAE, a specific animal model of MS (Chen et al., 2019). These findings from observational studies and experimental rodent models reveal the potential of *Ruminococcus* in promoting the onset of MS. Our MR estimates highlight the potential of targeting *Ruminococcus* in future MS therapeutic strategies. In addition, several studies have reported associations between *Anaerofilum* and neurocognitive deficits as well as depression (Barandouzi et al., 2020; Liu et al., 2022). Given the prevalence of neuropsychiatric disorders in MS patients (Compston & Coles, 2008; Dendrou et al., 2015), we hypothesize that *Anaerofilum* may increase the risk of MS, a hypothesis supported by our MR analysis. Several studies suggest that oral broad‐spectrum antibiotics may prevent or ameliorate MS (Mangalam et al., 2017; Shahi et al., 2020). Based on our findings, antibiotic interventions targeting *Ruminococcus* and *Anaerofilum* may be beneficial for MS patients. However, although these findings are promising, their use should be approached with caution and requires validation, given that antibiotics may alter more than just specific bacteria and the potential adverse effects of long‐term use.

To date, research on the association between MS and *Ruminoclostridium* remains limited. One study reported a significant decrease in *Ruminoclostridium* in Crohn's disease (El Mouzan et al., 2018), which is consistent with findings in an animal study of ulcerative colitis (Lian et al., 2022). These studies suggest that *Ruminoclostridium* may potentially reduce inflammatory responses. This partially explains the protective causal effect of *Ruminoclostridium* on MS observed in our analysis. It is worth noting that accumulated observational studies have suggested associations between different gut microbiota, such as *Bacteroides* and *Faecalibacterium*, and MS (Cantarel et al., 2015; Kujawa et al., 2023; Levi et al., 2021; Swidsinski et al., 2017). However, these associations were not replicated in our study and require further validation.

Accumulating evidence suggests that *Ruminococcaceae* may confer a reduced risk of MS. Differences in the abundance of *Ruminococcaceae* and other genera have been reported between the gut microbiota of patients with relapsing‐remitting MS (RRMS) and healthy controls (Navarro‐López et al., 2022). Tremlett et al. (2021) observed that MS patients had a reduced abundance of SCFA‐producing *Ruminococcaceae* in the gut compared to patients with monophasic acquired demyelinating syndrome. Another study investigating early‐onset childhood MS found a significantly reduced abundance of *Ruminococcaceae* compared to healthy controls (Tremlett et al., 2016). Horton et al. (2021) reported that higher levels of *Ruminococcaceae* were associated with a reduced risk of clinical relapse in pediatric‐onset MS (relapse hazard ratio = 0.45, 95% CI: 0.22–0.91). These findings suggesting an increased abundance of *Ruminococcaceae* as a protective factor against MS are consistent with our MR estimate (OR = 0.822, 95% CI: 0.688–0.982, *p* = .031). In addition, our study highlighted a causal effect of *Anaerotruncus* on a reduced risk of MS (OR = 0.772, 95% CI: 0.634–0.940, *p* = .010), although this contrasts with the results of a particular animal study (Bianchimano et al., 2022). In our study, these specific bacterial taxa, including *Ruminoclostridium*, *Ruminococcaceae*, and *Anaerotruncus*, can be considered probiotics. When administered in sufficient quantities to MS patients, they may increase levels of anti‐inflammatory mediators, suppress pro‐inflammatory factors, and benefit the host (Kujawa et al., 2023). Results from clinical trials and meta‐analyses support that probiotic supplementation may improve disease progression and psychiatric symptoms in MS patients (Jiang et al., 2021; Kouchaki et al., 2017; Mirashrafi et al., 2021). Therefore, the three bacterial taxa identified with potential protective causal effects in our study may serve as candidates for clinical trials to further determine efficacy.

The potential protective mechanisms of *Ruminococcaceae* and *Anaerotruncus* against MS might be associated with their role in increasing the production of butyrate in the gut (Bianchimano et al., 2022; Chin Fatt et al., 2023; Lee et al., 2023). Butyrate, acting as an inducer of Tregs (Haghikia et al., 2015), has been shown to promote oligodendrocyte differentiation, inhibit demyelination, and stimulate remyelination, reducing the risk of MS relapses and the emergence/extension of magnetic resonance imaging lesions (Chen et al., 2019; Horton et al., 2021). Furthermore, as a short‐chain fatty acid, butyrate can reduce visceral fat accumulation (He et al., 2023; Nie et al., 2020). Given the rising global prevalence of obesity and its identification as one of the risk factors for MS (Afshin et al., 2017; Thompson et al., 2018), harnessing butyrate‐producing *Ruminococcaceae* and *Anaerotruncus* may represent a promising avenue for the prevention and treatment of MS. In addition, *Ruminoclostridium* may relieve MS symptoms through its anti‐inflammatory effects (El Mouzan et al., 2018; Lian et al., 2022).

The enrichment analysis results suggest that intercellular communication within cell membranes mediates the regulatory effects of gut microbiota on MS risk. This supports a potential mechanism whereby dysbiosis of the gut microbiota may alter peripheral immune tolerance and promote the production of pro‐inflammatory mediators such as cytokines. The peripheral immune system communicates with the CNS via cytokines and other mediators, leading to neuroinflammation and immune responses in the brain and spinal cord regions (Amruta et al., 2021; Cosorich et al., 2017; Dendrou et al., 2015; Noto & Miyake, 2022). Pathway enrichment analysis reveals that dendritic cell development may be associated with gut microbiota involvement in MS regulation, supporting the notion that gut microbiota signals mediate intercellular communication among dendritic cells, contributing to an unbalanced immune environment (Correale et al., 2022; Parodi & Kerlero de Rosbo, 2021). Furthermore, the pathway analysis suggests that oxidative stress may also be involved in these biological processes, consistent with the hypothesis that *Ruminococcus* induces inflammation and immune responses in the CNS (Azzouz et al., 2019; Cantarel et al., 2015).

This study has several strengths. First, we used the MR method to systematically assess the causal relationship between gut microbiota and MS, effectively mitigating confounding and reverse causality. Second, we selected robust genetic tools as IVs and used multiple MS databases for comparison, followed by collaborative analysis. In addition, a series of sensitivity analyses confirmed the absence of heterogeneity or horizontal pleiotropy, underscoring the robustness of our statistical evaluations.

However, our study has several limitations. First, while the GWAS data are mainly derived from European populations, our findings may not be fully generalizable to other ethnic groups. Future investigations should examine the causal relationship in more specific populations. Second, we used a Bonferroni correction for multiple comparisons, which may be conservative (Zeng et al., 2023). Third, due to the lack of subtype‐specific MS data, we could not perform subgroup analyses, such as exploring the causal effects of different gut microbiota on RRMS or progressive MS (PMS). Given that RRMS accounts for the majority of MS cases (approximately 85%), we can surmise that the effects of RRMS may overshadow those of PMS in our current MR study. This suggests that our results may be more biased toward an assessment focused on RRMS. Importantly, although currently approved drugs for MS can reduce relapses, they do not necessarily stop the progression of neurodegenerative changes (Correale et al., 2017; Dendrou et al., 2015). This suggests that more targeted stratification of patients, particularly those with PMS, may have promising therapeutic potential.

In summary, our results suggest that out of 211 microbes, five are potentially associated with either an increased or decreased risk of MS. These findings advance our understanding of the mechanisms by which gut microbiota mediate the onset of MS and offer potential therapeutic avenues.

## AUTHOR CONTRIBUTIONS

**Dongren Sun**: Software; data curation; methodology; investigation; validation; formal analysis; resources; visualization; writing—original draft; writing—review and editing. **Yangyang Zhang**: Methodology; validation; visualization; investigation; writing—original draft; software; formal analysis; data curation; resources; writing—review and editing. **Rui Wang**: Methodology; software; data curation; visualization; writing—review and editing. **Qin Du**: Methodology; visualization; writing—review and editing; software; data curation. **Ziyan Shi**: Methodology; visualization; writing—review and editing; software; data curation; funding acquisition. **Hongxi Chen**: Methodology; visualization; writing—review and editing; software; data curation. **Xiaofei Wang**: Funding acquisition; conceptualization; writing—review and editing; resources; supervision; validation. **Hongyu Zhou**: Conceptualization; funding acquisition; writing—review and editing; supervision; resources; validation.

## CONFLICT OF INTEREST STATEMENT

The authors declare no conflicts of interest.

### PEER REVIEW

The peer review history for this article is available at https://publons.com/publon/10.1002/brb3.3593.

## Supporting information

Supporting Information

## Data Availability

Data underpinning the findings of this research can be found within the article and its supplementary materials.
